# Feasibility of Stepwise Technology‐Based Audiometry With Rapid Results (STARR) Protocol in Minnesota Elementary Schools

**DOI:** 10.1002/ohn.1192

**Published:** 2025-03-10

**Authors:** Autefeh Sajjadi, Morgan McBride, Kimberly Guettler, Stephanie Janasko, Staci House, Cesley Bergsten, Nobles Antwi, Madeline Marker, Soorya Todatry, Stacey Rabusch, Rebecca Maher, Brianne Roby, Abby C. Meyer, Andrew Redmann, Sivakumar Chinnadurai, Asitha D.L. Jayawardena

**Affiliations:** ^1^ Department of Otolaryngology–Head and Neck Surgery University of Minnesota Minneapolis Minnesota USA; ^2^ Department of Otolaryngology–Head and Neck Surgery University of Minnesota Medical School‐Twin Cities Minneapolis MN USA; ^3^ Edina Public Schools Edina Minnesota USA; ^4^ Children's Minnesota Research Institute Minneapolis Minnesota USA; ^5^ Department of Pediatric ENT and Facial Plastic Surgery Children's Minnesota, Pediatric ENT and Facial Plastic Surgery Minneapolis Minnesota USA

**Keywords:** disparities, early hearing identification, hearing screening, pediatric hearing loss

## Abstract

**Objective:**

This study aims to understand the utility of a stepwise technology‐based audiometry with rapid results (STARR) school screening protocol.

**Study Design:**

A prospective cohort study.

**Setting:**

Six elementary schools in a single school district in Minnesota.

**Methods:**

Students at 6 elementary schools in Minnesota participated in the STARR protocol and underwent initial technology‐based hearing screening, followed by additional comprehensive automated audiometry with insert earphones and point‐of‐care otoscopy if they were referred. Results were reviewed by an otolaryngologist remotely, and parents received treatment recommendations based on these findings.

**Results:**

A total of 454 (81% of eligible) students were screened and 27 students (5.9%) referred. On average, the initial screening took 55 seconds (standard deviation [SD] = 22) for those who passed and 116 seconds (SD = 55) for those who were referred. Comprehensive audiometry screening took 163 seconds (SD = 27) for those who passed and 252 seconds (SD = 100) for those who referred. A team of 6 screeners could screen a class of 30 students in 30 minutes. The total number of nursing encounters required to ensure a student saw a provider after a referral was reduced using the STARR protocol (2.47 encounters per referral) compared to traditional audiometric screening (3.39 encounters per referral) (*P* < .01).

**Conclusion:**

The STARR protocol is a feasible and efficient method of screening in public schools that can reduce false referral rate, provide parents with more information at the point of referral, and reduce nursing burden.

**Implications for Practice:**

Technology‐based hearing screenings should be considered in school settings as a means to provide more patient and family‐centered hearing health care. Further research is necessary to understand how the STARR protocol influences loss to follow‐up rates after failed hearing screening.

School hearing screening is a critical public health intervention that facilitates the identification of pediatric hearing loss occurring after newborn hearing screening or hearing loss not detected on newborn hearing screening.^1^ The impact of undetected pediatric hearing loss is life‐changing and can lead to speech and language delays, decreased school performance, and increased risk of school dropout.^2^ The World Health Organization estimates that the global cost of untreated hearing loss in the educational system alone is $3.9 billion.^3^

The prevalence of pediatric hearing loss that occurs after the newborn period increases with time.^4^ Up to 14% of school‐aged children have transient hearing loss in one or both ears.^5^ In the United States, the prevalence of permanent loss, which at the age of 6 years is only 0.6%, increases to about 1% in school‐age children and up to 5% in adolescents.^6^, ^7^ This can be due to progressive congenital hearing loss, congenital cytomegalovirus, and/or a number of acquired risk factors including recurrent acute otitis media, chronic otitis media, trauma, exposure to ototoxic medication, and noise exposure.^4^, ^8^ In fact, nearly 1 billion children are at risk of hearing loss due to unsafe listening practices.^9^

Hearing loss that occurs after the newborn hearing screen is typically identified on school‐based hearing screening.^10^ Up to 50% of 9‐year‐olds with educationally significant hearing loss pass their newborn hearing screening.^11^ School hearing screening has been shown to be a cost‐effective means to identify hearing loss in school‐age children.^12^ However, despite the clear benefits of school‐based screening, there remains enormous variability in school screening protocols globally.^13^ In fact, only 66% of US schools perform any school‐based hearing screening whatsoever, despite national recommendations that school screenings be performed regularly.^13^ High rates of false positive testing reduce the cost‐effectiveness of school‐based hearing screening.^12^ Additionally, existing screening programs demonstrate loss to follow‐up after referral ranging from 35% to 90%, which has been universally identified as a targeted area for improvement.^13^ Loss to follow‐up has been shown to be at least in part due to a lack of parental awareness regarding the medical significance of the hearing screening result.^14^ Despite these data, there have been no major innovations in school‐based hearing screening in the past several decades.^13^

A number of technological innovations have improved access to hearing health care over the past decade with a focus on the utilization of existing capital (Internet, smartphones, etc.) to improve access.^15^ Some of these technologies have been piloted in the school setting with variable success in low‐resourced areas of the globe.^16^, ^17^ The utility of technology‐based hearing screening includes a lower rate of false positive referrals and the incorporation of more information into the point of care to improve parental understanding.^16^, ^18^ To date, no technology‐based school hearing screening program have been implemented with success in US public schools. This study describes a prospective technology‐based school hearing screening protocol that aims to provide more hearing‐specific information to parents at the time of referral, therefore providing more patient‐ and family‐centered hearing health care.

## Methods

### Study Design and Setting

This is a prospective cohort study in which first graders across 6 elementary schools in a single school district (Edina Public Schools) in Minnesota were enrolled. The student body in this district is 52% male. The majority of students in this district are white (67.6%), followed by black/African American (10.3), Asian (9%), Hispanic/Latino (6.9%), or multiple races (6%). Eight percent of students are eligible to participate in the federal Free and Reduced Lunch Program, and 5.6% of students are English language learners (students learning English as a second language).

Hearing screening was implemented in the school calendar year 2023 to 2024 in a single day at each elementary school. A quiet testing location within the school was identified before screening at each location. The screening was performed on a class‐by‐class basis to minimize disruption to the elementary school curriculum. Screenings were performed in groups of up to 5 children at the same time, in the same room, to maximize efficiency. A lead screener would provide instruction to all 5 students simultaneously once they entered the quiet room. Students were then paired with a screener who performed the screening. Students were instructed to remain quiet until all students in the room had completed their testing. Children who passed the screening were then escorted out of the room. If the child referred their initial screen, they were taken to a separate room for more comprehensive hearing screening and point of care otoscopy. Otoscopy was performed by either a medical student, otolaryngology resident, or otolaryngologist. Screeners had a variety of audiometric screening backgrounds and included otolaryngologists, audiologists, school nurses, medical students, and research assistants. All testing was administered in English by the research staff.

Data were compared to the students from the year before initiating technology‐based screening (2022‐2023), which was done using a traditional audiometer playing pure‐tone (500 Hz at 25 dB, 1000 Hz at 20 dB, 2000Hz at 20 dB, and 4000 Hz at 20 dB) frequencies by school nursing staff/volunteers. Children who failed in the traditional cohort were rescreened either the same day and/or 2 weeks later until they passed or were ultimately referred. Both the traditional cohort and the technology‐based cohort were performed in a quiet testing location in the school. A “nursing encounter” was defined as an effort by the nurse to contact the parent (via phone, text, etc.) to provide the results of the hearing screening. A closed referral was defined as a child who successfully participated in an encounter with a pediatrician, audiologist, or otolaryngologist regarding their hearing health. The referral reduction rate is the specific reduction in true referrals obtained once the comprehensive automated audiometry is utilized on all children who refer to the initial screen (HearScreen^TM^).

This study was approved by Children's Minnesota Institutional Review Board (2023‐043). Written consent forms were sent to all parents before testing, and only children whose parents performed written consent were allowed to participate. Children aged 7 years and older were also required to verbally assent to participating and those who did not verbally assent were excluded from the study. Additionally, any child who demonstrated discomfort or voiced a desire for exclusion was excluded from the study and could opt out at any time. Children who were not able to participate secondary to behavioral concerns (autism, etc.) were also excluded if they could not tolerate the testing.

### Stepwise Technology‐Based Audiometry With Rapid Results (STARR) Protocol

Initial screening data were collected using Android‐based cellphones (Samsung Galaxy A04e) equipped with HearX HearScreen applications (HearX Group). Each phone was paired with a pre‐calibrated Sennheiser HD280 Pro headphone. Comprehensive automated audiometric testing was collected via an Android‐based tablet (Samsung Tab A7) equipped with HearX HearTest applications with IP30 insert earphones. Students were given a unique identifier number to protect their identity in terms of data breach. Test results were immediately stored on the phones/tablets and automatically uploaded wirelessly to a Health Insurance Portability and Accountability Act (HIPAA) compliant web‐based patient chart once the phones had Internet connectivity. Only the patient's unique identifier number tied their hearing data to their chart in the cloud.

**Figure 1:**
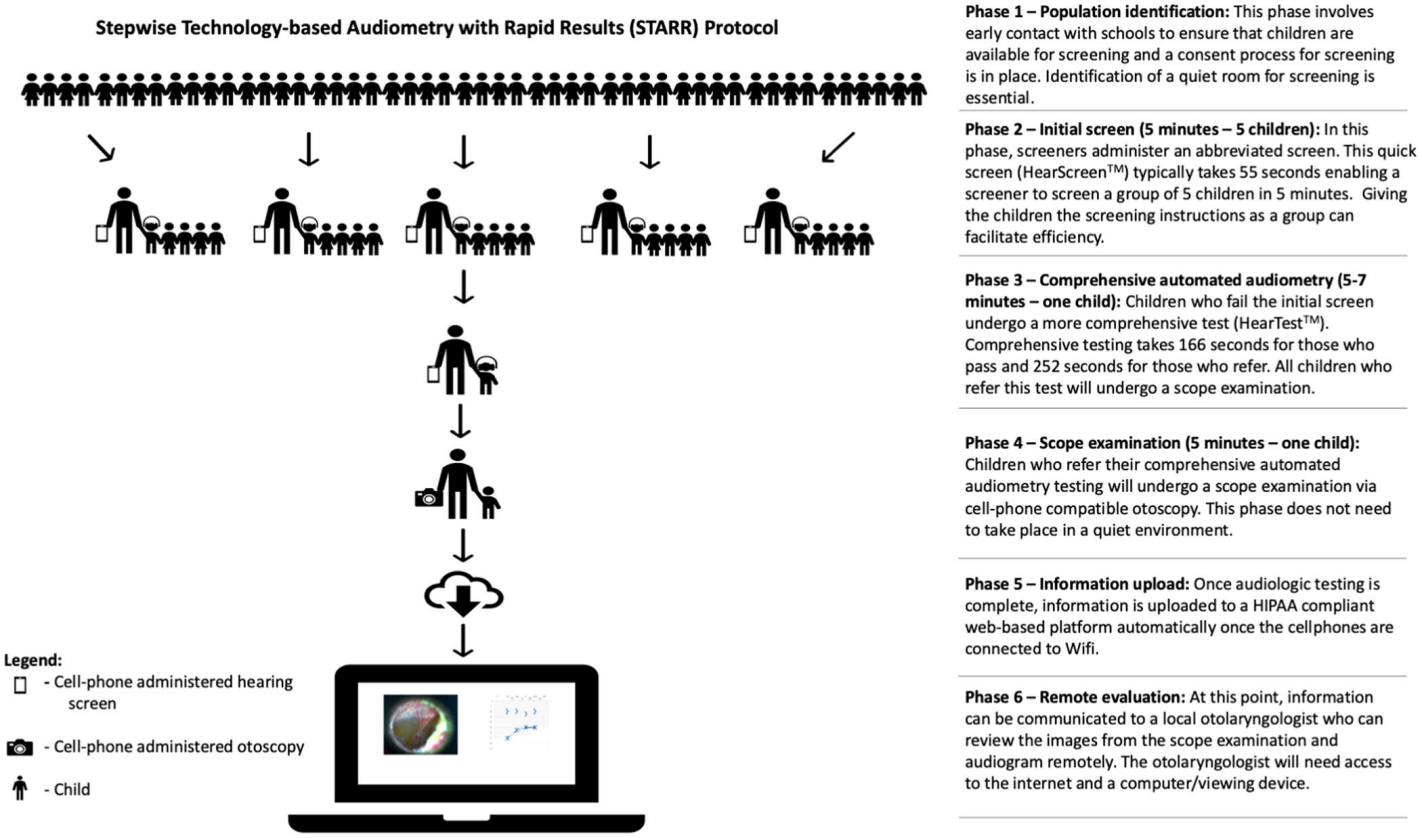
STARR protocol.

This study utilizes a multi‐tiered hearing screening algorithm (Figure 1) that has previously been described by the senior author.^16^ First, all children underwent a preliminary audiologic screening (HearScreen^TM^). The HearScreen^TM^ application has previously been validated as achieving similar sensitivity/specificity and referral rates compared to traditional audiometers in the school settings.^19^, ^20^, ^21^ For this screen, subjects were presented with pure‐tone stimuli separately for each ear at 500, 1000, 2000, and 4000 Hz frequencies. All preliminary screen stimuli were presented at 25 dBHL. To participate, children were instructed to raise their hands when they perceived a tone. Children were “conditioned” how to participate in the screening via the conditioning feature on the cell phone software, which allows the tester to play a loud sound on command to facilitate test understanding.

Children were positioned facing away from the screeners during the testing so they could not see the phones or the instructor during testing. During the examination, if the child raised their hand after a tone was presented, the tester would mark “correct” for that frequency. If a student did not raise their hand after a tone, the tester would mark “incorrect” for that frequency, and move on to the next frequency. If the participant was marked “incorrect” for a frequency, the software was programmed to retest this frequency at the end of the screen. A “pass” for the preliminary screen indicated a raised hand for every presented frequency in both ears, while a “fail” indicated at least 1 frequency that was incorrect. The software was programmed to re‐test any frequency that the student did not hear at the first presentation. The screening platform has a clinically validated background noise monitoring system that has been previously described.^22^ The ambient noise levels during testing were recorded and testing was automatically paused if ambient noise levels were deemed too high by the software.

Any subject who failed the first stage of screening proceeded to the second stage of more comprehensive testing (HearTest™). This more comprehensive automated audiometric test was administered using the tablet, which included insert earphones that provided better sound attenuation (40‐50 dB).^23^ Comprehensive screening not only performed a more thorough test battery but also reduced the effect of ambient noise, which prior studies have found to be a major barrier to efficient screening.^16^ False positives are tracked through the testing software as not all prompts result in a sound being played. Additionally, the pre‐tone waiting period is varied so the student does not become conditioned to respond after a certain period of time. The tester is notified of an unreliable test if the student has increased false positive responses. Stimuli presented for comprehensive screening included 500, 1000, 2000, and 4000 Hz for each ear. Unlike the preliminary test, comprehensive automated audiometry obtained hearing thresholds in the traditional Hughson‐Westlake method by bracketing the threshold in an up‐down fashion in an automated fashion using the phone software. For each ear, a pure‐tone average was automatically calculated by the application (average of the thresholds at 500, 1000, and 2000 Hz frequencies). Similar to the preliminary screen, stimuli were presented at 25 dBHL and above. A “fail” for the comprehensive examination was classified by a pure‐tone average of over 25 dBHL. Comprehensive testing was done on the same day as the initial screening.

Failure on comprehensive automated audiometry prompted otoscopy to rule out transient causes for hearing loss—the third phase of testing. The endoscopic (HearScope^TM^) examination implements the use of camera software in addition to an endoscopic camera specifically designed to capture images of the external ear canal and tympanic membrane. Otoscopic images were stored along with audiologic testing results on a secure server in the cloud.

**Figure 2:**
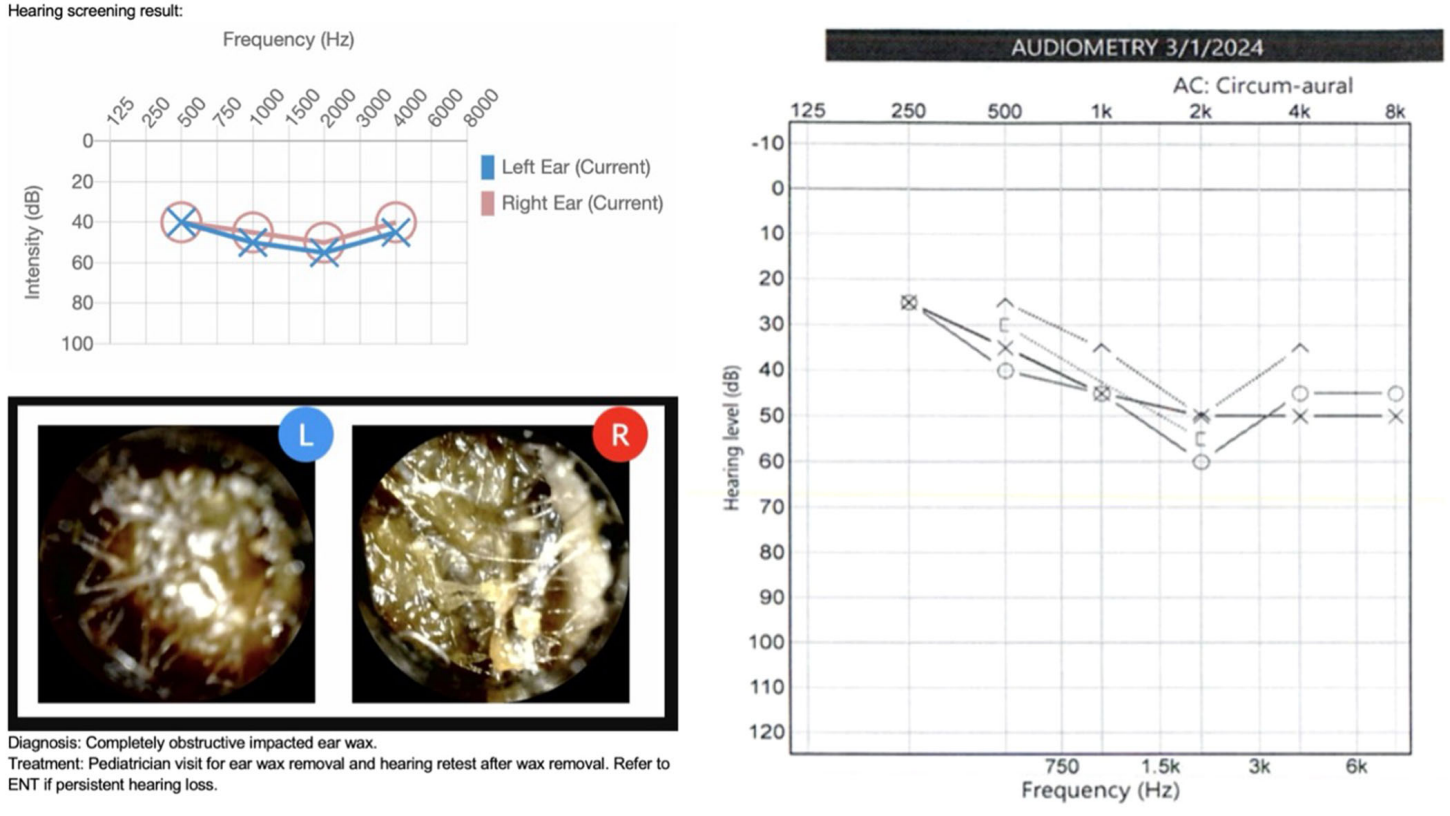
STARR protocol referral sheet (left) compared to formal conventional audiometry in soundbooth. The student brought STARR protocol referral sheet to the appointment and was identified with SNHL and fit with hearing aids. SNHL, sensorineural hearing loss.

Upon completion of this testing battery, the children were sent home with a paper document which had a printed audiogram and otoscopy with an interpretation of their findings (ie, unilateral hearing loss with a tympanic membrane perforation). This also included a recommendation for the next steps (ie, rescreen in 2 weeks, follow‐up pediatrician, follow‐up otolaryngology/audiology, etc.). The recommendation was made by an otolaryngologist who reviewed the audiogram and images remotely. An example of a STARR protocol referral sheet (compared to conventional audiometry on the same student after they presented to otolaryngology) is shown in Figure 2. Schools used their local translational services to translate this into the student's home language to give to parents.

### Data Analysis

Data collected in the field were uploaded from smartphone devices to the HIPAA‐compliant cloud database. These data were then exported and analyzed using Microsoft Excel (Redmond). Audiometric data herein are presented according to the 1995 AAO‐HNS consensus guidelines. Continuous variables were reported as means with standard deviations when normally distributed and medians with ranges when not normally distributed. Student and paired *t*‐tests were used to compare traditional and technology‐based audiometric testing means assuming normally distributed data with all tests 2‐sided.

## Results

**Figure 3:**
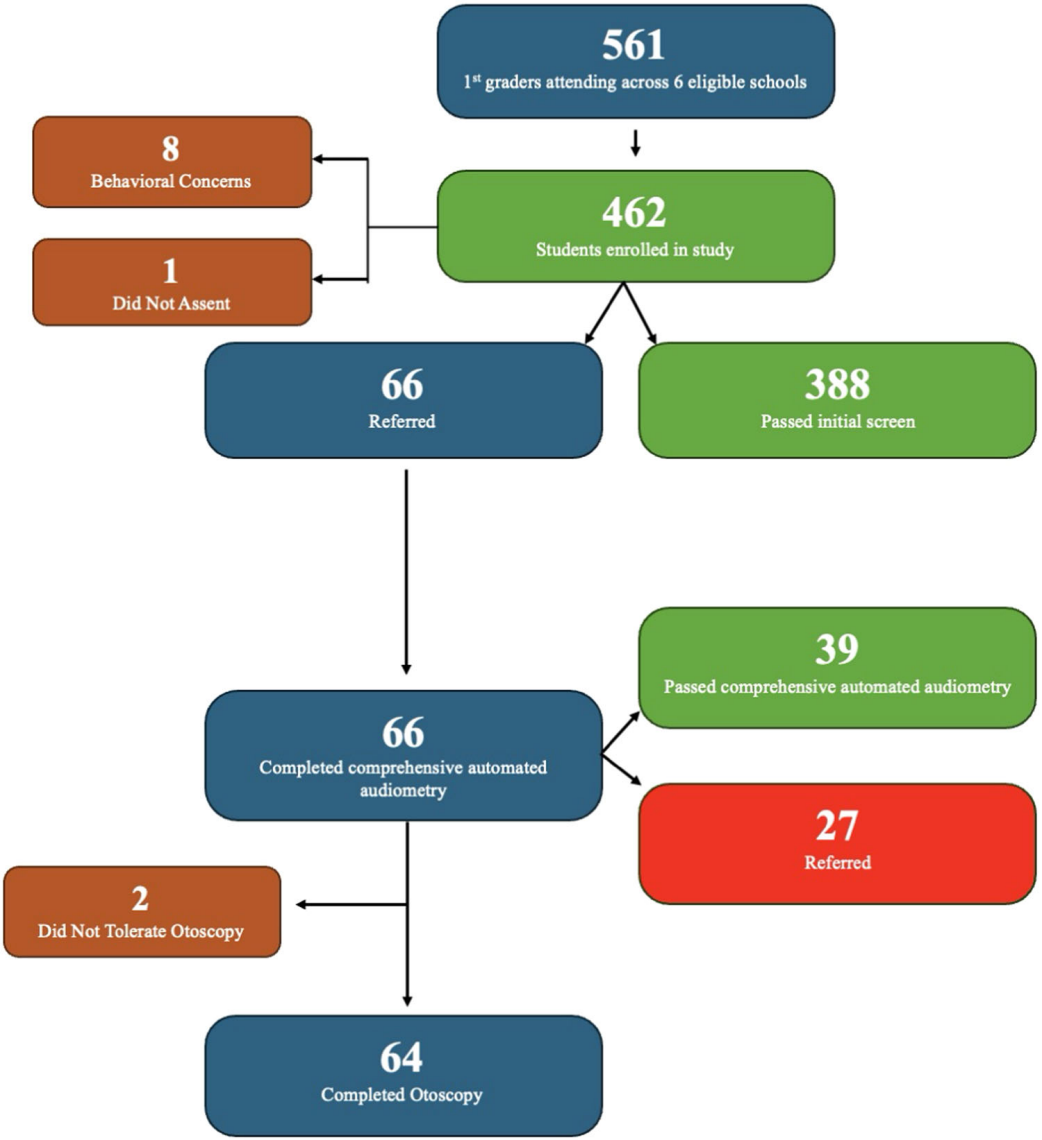
Flowchart demonstrating the progression of students enrolled in the study from the initial screen to comprehensive automated audiometry and otoscopy.

There were 561 first‐grade students enrolled across the 6 schools, 462 parents provided informed consent to participate in the study, 8 students were not able to participate secondary to behavioral concerns, and 1 student did not provide verbal assent in addition to their parental consent. Therefore, 454 students (81% of eligible) proceeded with the initial screen: 388 passed (85.5%) and 66 (14.5%) were referred for further testing. Of the 66 students who were initially referred, 39 students (59.1%) passed the more comprehensive testing and 27 (40.9%) were ultimately referred (Figure 3). Therefore, comprehensive automated audiometry had a referral reduction rate of 60.6%.

### Automated Audiometry and Otoscopy Data

**Figure 4:**
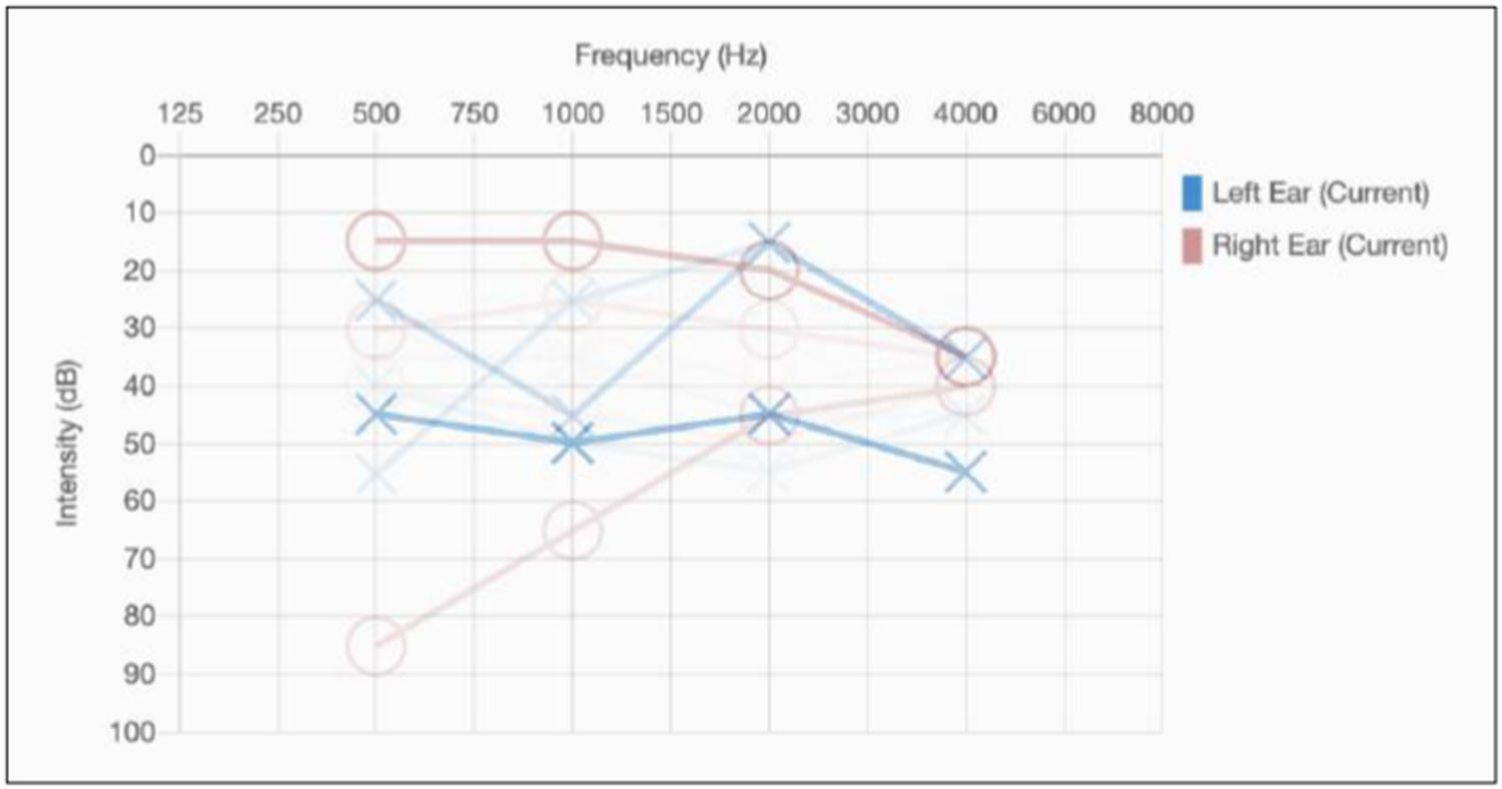
Comprehensive automated audiometric findings from all 27 students who were referred using STARR protocol. Fourteen students (51.9%) were found to have unilateral mild hearing loss, 2 students (7.4%) with unilateral moderate hearing loss, 7 (25.9%) with bilateral mild hearing loss, 1 (3.7%) with bilateral moderate hearing loss, and 3 (11.1%) with asymmetric hearing loss (1 side mild and 1 side moderate).

Audiograms from the 27 students who were referred are displayed in Figure 4. Fourteen students (51.9%) were found to have unilateral mild hearing loss, 2 students (7.4%) with unilateral moderate hearing loss, 7 (25.9%) with bilateral mild hearing loss, 1 (3.7%) with bilateral moderate hearing loss, and 3 (11.1%) with asymmetric hearing loss (1 side mild and 1 side moderate). Twenty‐five students (92.6%) proceeded with otoscopy as 2 were unable to tolerate the otoscope insertion. Among the 25 students, 12 (48%) had evidence of effusion, 5 (20%) with cerumen, 1 (4%) had retraction pockets, and 6 (24%) were normal. Eight students with effusion passed their follow‐up hearing test 2 weeks later resulting in a total of 19 total referrals.

### Feasibility Data

On average the initial screen took 55 seconds (SD = 22) for those who passed, and 116 seconds (SD = 55) for those who referred, as it would repeat the failed frequencies (*P* < .01). Comprehensive automated audiometry took 163 seconds (SD = 27) for those who passed and 252 seconds (SD = 100) for those who referred (*P* < .01). Including downtime between testing, a team of 6 screeners (5 running basic screening and 1 running comprehensive automated audiometry and otoscopy) could screen a class of 30 students in 30 min. This is similar to the timing of traditional hearing screening methods according to per school nursing staff.

### Closing a Referral

The number of students that referred the year prior with the utilization of traditional hearing screening (18 referrals) was comparable to the number of students who were referred using technology‐based hearing screening (19 referrals) (*P* = .867). The total number of nursing encounters required for a referral to be closed was reduced using technology‐based hearing screening (2.47 encounters per referral) compared to traditional audiometric screening (3.39 encounters per referral) (*P* < .01). This is a 27% reduction and time savings for nursing staff. Twelve children required 3 or more encounters to close the referral with traditional screening while only 7 required 3 or more encounters to close a screening using technology‐based screening (*P* = .07). The rate of follow‐up with a hearing health care provider after the initial nursing point of contact increased from 10% using traditional school screening methods to 60% with the STARR protocol.

## Discussion

Technology‐based hearing screenings are feasible, efficient, and effective in identifying hearing loss in school setting and can reduce the required nursing encounters necessary to ensure a child receives the hearing health care they need. Referral rates are comparable to traditional hearing screening using standard methodologies; however, parents receive significantly more information than traditional methods at the point of care. A picture of an audiogram and a child's eardrum combined with a clinician's recommendation for follow‐up is significantly more information than prior standardized forms which were sent home and stated that the child was referred on their hearing screening. The extra data sent home with children in the STARR protocol facilitate a more patient and family‐centered approach to health care. Parents are more data‐driven in their educational interactions today than they ever have been, including in audiologic health care.^24^ However, further data are needed to understand this trend across different minority groups.^24^

The extra objective data points provided to parents regarding their child's hearing may facilitate engagement in a more meaningful way than a sheet of paper that says “refer.” This is particularly important for parents who may not perceive their child to have a hearing loss. School screening and parental concern are, in fact, the mainstay of identification of hearing loss that isn't identified by the newborn hearing screen.^10^ The easier time that nursing staff had to close a referral suggests that parental engagement in hearing health care is increased using the STARR protocol. A substantial 81% of eligible students had parents sign consent to participate in the STARR protocol indicating strong parental engagement and is encouraging regarding the feasibility of implementing such hearing screening programs in educational settings.

This study is limited in that the research team performed all screenings per our IRB protocol as this was a pilot test of this technology in this district. Therefore, it is unclear if this is a sustainable intervention that the school and school staff can run independently without the resources of a study team. Further research is essential to understand the sustainability of technology‐based hearing screening in the school setting. There may also be a participation bias in that only parents who believed their children could participate in such a screening tool provided consent. Additional work is needed to understand the feasibility of this study when prospective parental consent is not a barrier and all children can be enrolled. This study was done in a somewhat homogenous population in Minnesota, and further research is necessary to expand to more diverse school districts across the country to better understand the generalizability of this project. The school district in which this was performed has a lower incidence of free and reduced lunches than other districts, suggesting that it is a higher resourced district. More work is necessary to understand the utility of this program in low‐resourced settings. Future research should also focus on understanding the longitudinal hearing health of those identified using technology‐based hearing screening. Hearing screenings were performed in isolated rooms that did have ambient noise (ie air conditioning vents and school alarms) and thus potential accuracy of hearing screenings could be affected; however, the utilization of more comprehensive automated audiometry was beneficial in reducing the rate of false referrals as well as software in the smartphones, which paused testing during periods of high ambient noise.

Although the STARR protocol requires upfront capital costs for new hearing screening equipment, this investment may be worth it when considering the increased information at the point of care provided to families and the reduction in nursing burden for an already overworked workforce within the public school system. The largest targetable gap in school screening was the aforementioned 35% and 90% loss to follow‐up.^13^ Longitudinal data on how the STARR protocol influence this follow‐up rate at a larger scale is imperative. School budgets are inherently limited; therefore, more research is needed to better understand the feasibility, scalability, and sustainability of the STARR protocol in public schools.

## Implications for Practice

The utilization of the STARR protocol is a safe, effective, and efficient method of screening in public schools that can both reduce the burden on nursing staff and provide parents with more information at the point of care to facilitate referral. This more patient and family‐centered approach should help reduce the loss to follow‐up seen after a failed hearing screen at the school level. Further research is necessary to study this intervention at scale and in settings of varied socioeconomic diversity.

## Author Contributions

**Autefeh Sajjadi**, study design, data acquisition, analysis, presentation, drafting and editing of manuscript; **Morgan McBride**, data acquisition, data analysis, drafting and editing manuscript; **Kimberly Guettler**, study design, data acquisition, data analysis, drafting and editing manuscript; **Stephanie Janasko**, study design, data acquisition, data analysis, drafting and editing manuscript; **Staci House**, study design, data acquisition, data analysis, drafting and editing manuscript; **Cesley Bergsten**, study design, data acquisition, data analysis, drafting and editing manuscript; **Nobles Antwi**, data acquisition, data analysis, drafting and editing manuscript; **Madeline Marker**, data acquisition, data analysis, drafting and editing manuscript; **Soorya Todatry**, data acquisition, data analysis, drafting and editing manuscript; **Stacey Rabusch**, study design, data acquisition, drafting and editing manuscript; **Rebecca Maher**, study design, data acquisition, drafting and editing manuscript; **Brianne Roby**, study design, drafting and editing manuscript; **Abby C. Meyer**, study design, drafting and editing manuscript; **Andrew Redmann**, study design, drafting and editing manuscript; **Sivakumar Chinnadurai**, study design, drafting and editing manuscript; **Asitha D.L. Jayawardena**, study design, data acquisition, study analysis, presentation, drafting and editing of manuscript.

## Disclosures

### Competing interests

None.

### Funding source

This study was funded by the Minnesota Lions Hearing Grant.
